# Acute methaemoglobinaemia due to ingestion of nitrobenzene (paint solvent)

**DOI:** 10.4103/0019-5049.63635

**Published:** 2010

**Authors:** Hema Saxena, Anand Prakash Saxena

**Affiliations:** Anesthesia Intensive Care and Pain Relief, SGRR Institute of Medical and Health Sciences, Dehradun, Uttarakhand, Asha Hospital, Dehradun, India

**Keywords:** Acute methaemoglobinaemia, methylene blue, nitrobenzene poisoning

## Abstract

A case of acute poisoning with nitrobenzene is presented where clinical evaluation and timely management, with repeated intravenous methylene blue helped to save a life. It is important to take care of the secondary cycling of nitrobenzene from body stores in patients presenting late, after heavy exposure.

## INTRODUCTION

Acute poisoning with nitrobenzene causing significant methaemoglobinaemia is uncommon but life threatening emergency. Early aggressive management of severe poisoning, strongly suspected on clinical grounds may change the outcome of a patient.

## CASE REPORT

A 16-year-old, unconscious female presented to Emergency with cyanosis and a greyish-brown hue, laboured respiration of 26/min, BP 150/100 mm of Hg, pulse rate 160/min, pupils with sluggish reaction, and SpO_2_ of 60% on air. Her chest was clear. Immediate intubation and ventilation with 100% oxygen and 6 cm PEEP improved the SpO_2_ to 84% only. There was a history of severe pain in the abdomen, nausea, vomiting, and dizziness, which was self-treated with tablet metoclopromide, about eight hours prior to unconsciousness. The patient was referred by a local practitioner who started an IV infusion and administered a shot of mephentermine and hydrocortisone after recording a BP of 40 mm Hg. History of ingestion of any drug was strongly denied by the companion. An urgent ultrasound abdomen ruled out any abdominal catastrophe and showed a generalized mild-to-moderate hepatic inflammation. Blood samples drawn for ABG had a chocolate brown colour, which did not improve on exposure to 100% oxygen and showed compensated metabolic acidosis [Table 1]; X-ray of the chest and ECG were within normal limits, and WBC and liver enzymes were slightly raised. Serum creatinine and electrolytes were within normal range [Table 2]. A clinical diagnosis of severe acute methaemoglobinaemia of unknown origin was made.

**Table 1:** ABG

	pH	PaCO_2_(mm hg)	PaO_2_(mm Hg)	HCO_3_	SpO_2_%	SpO_2_%
At admission	7.41	30	57	20.2	80	60
6 hours	7.42	30	167	18.5	94	84
24 hours	7.42	33	112	23.4	92	88
48 hours	7.43	35	116	24.8	96	90

**Table 2:** Investigations

	TLC	DLC	SGOT (IU/lit)	SGPT (IU/lit)	INR	UREA	Creatinine	Na	K	Hb g%
At admission	26,500	P90,L9E1	92	106	1.37	26	0.9	137	3.8	11.5
24 hours	16,500	P90,L9,E1	194	80	1.38	23	0.7	139	3.4	9.9
48 hours	19,900	P93,L6,EI	195	124	1.41	22	1.0	142	3.9	8.8
3^rd^ day	11,500	P81,L17,E2	-	-	1.32	23	0.69	139	3.2	10
5^th^ day	12,700	P82,L16,E2	139	73	1.05	-	0,7	137	3.8	9.3
7^th^ day	11,500	P90,L8,E2	106	124		-		-		8.3
9^th^ day	7,700	P72,L25E3	106	109	1,08	-		-		7.1

Five millilitres (50 mg) of methylene blue (prepared as 1% sterile solution) and ascorbic acid 500 mg were given IV. This improved her SpO_2_ to 92%, which dropped after about three hours, when 30 mg IV methylene blue was repeated. Intravenous Vitamin K, 10% dextrose, and an antibiotic were also added. Urine output was maintained above 100 ml/hour with proper hydration and frusemide, maintaining a normal central venous pressure (CVP).

She became conscious after six hours with a stable BP of 120/80 and HR of 120/minute. SpO_2_ was again 84% with ABG showing a saturation of 94%. IV methylene blue (30 mg) improved SpO_2_ to 97% over the next 15 minutes, only to return to 85% in the next three hours, with a similar response to another dose. Two units of fresh blood were transfused and this improved the SpO2 to 92 – 94%. She was extubated at 48 hours and maintained on a *continuous positive airway pressure* (CPAP) mask, with ABG showing a saturation of 93% and a PaO_2_ of 112, when she admitted to having consumed 250 ml of paint solvent (equivalent to 12.5 g of nitrobenzene). The SpO_2_ dropped back to 87% in next 12 hours.

With this waxing and waning picture of symptoms, six hourly gastric lavage with charcoal, purgation with polyethylene glycol (peg leg), intravenous methylene blue every six hours (three days) and then orally up to seven days, and IV ascorbic acid (500 mg) per day up to six days, was prescribed, till the smell of bitter almonds disappeared completely from the stools.

She improved rapidly after the seventh day with an SpO_2_of 90% on room air. International normalized ratio decreased to 1.08, although the liver enzymes were raised. She was discharged on the ninth day on oral iron, folate, ascorbic acid, and liver enzyme supplements and breathing exercises.

## DISCUSSION

Nitrobenzene, a pale yellow oily liquid, with an odour of bitter almonds is used as an intermediate in the synthesis of solvents, like paint remover. The first report of nitrobenzene poisoning came in 1886[1] and subsequent fatality reports followed.[1,2] Intoxication can be accidental or suicidal, or the side effect of some drugs, including metoclopromide.[2] Accidental toxicity can occur in patients consuming well water with dangerously high levels of nitrites and nitrates.[3] The lethal dose is reported to range from 1 g to 10 g, by different authors.[4,5] A review of published reports does not provide any consistent reports regarding fatalities and dose of ingestion.[5] The toxic effects after ingestion are due to the rapid development of methaemoglobinaemia,[4] a condition in which the iron within the haemoglobin is oxidized from the ferrous (Fe^2+^) state to the ferric (Fe^3+^) state, resulting in the inability to transport oxygen and causes a brownish discolouration of the blood.[3] Once formed, methemoglobin can be reduced enzymatically either via an Adenine dinucleotide (NADH)-dependent reaction, catalysed by cytochrome b5 reductase, or an alternative pathway utilizing the nicotine adenine dinucleotide phosphate (NADPH)-dependent methemoglobin reductase system.[2]

Acute intoxication is usually asymptomatic up to the level of 10 – 15% of methemoglobin, showing only cyanosis. Beyond 20%, headache, dyspnea, chest pain, tachypnea, and tachycardia develop. At 40 – 50%, confusion, lethargy, and metabolic acidosis occur leading to coma, seizures, bradycardia, ventricular dysrythmia, and hypertension. Fractions around 70% are fatal. Anemic or G6PD-deficient patients suffer more severe symptoms.[2,4] Leukocytosis has been reported, with relative lymphopenia.[5]

Other effects include hepatosplenomegaly, altered liver functions, and Heinz body haemolytic anaemia.[2,6] Nitrobenzene is metabolized to p-nitrophenol and aminophenol and excreted in urine, up to 65%, and in stools up to 15%, after five days of ingestion. Liver stomach, blood, and brain may act as stores and release it gradually.^6^

Clues for diagnosis include a history of chemical ingestion, the characteristic smell of bitter almonds, persisting cyanosis on oxygen therapy without severe cardiopulmonary disease, low arterial oxygen saturation, with normal ABG (calculated) oxygen saturation. Dark brown blood that fails to turn bright red on shaking, which suggests methaemoglobinaemia and this is supported by the chocolate red colour of dried blood. Presence of nitrobenzene compounds may be confirmed spectrophotometrically and estimated by the butanone test of Schrenk,[1] methemoglobin levels in the blood, and urinary presence of p-nitrophenol and p-aminophenol.[1,6,7]

Recommended treatment is based on the principles of decontamination and symptomatic and supportive management. Methylene blue is the antidote of choice for the acquired (toxic) methaemoglobinaemia. It is an exogenous cofactor, which greatly accelerates the NADPH-dependant methemoglobin reductase system and is indicated if the methemoglobin levels, which are more than 30%.[4] It is administered intravenously at 1 – 2 mg/kg (up to 50 mg dose in adults,) as a 1% solution over five minutes; with a repeat in one hour, if necessary. Methylene blue is an oxidant at levels of more than 7 mg/kg, and therefore, may cause methaemoglobinaemia in susceptible patients. It is contraindicated in patients with G6PD deficiency, because it can lead to severe haemolysis. Ascorbic acid is an antioxidant that may also be administered in patients with methemoglobin levels of more than 30%.[8] In recent studies, N-acetylcysteine has been shown to reduce methemoglobin, but it is not yet an approved treatment for methaemoglobinaemia.[8] Exchange transfusion is indicated in severe cases.[4,8] Hyperbaric oxygen is reserved only for those patients who have a methemoglobin level > 50% or those who do not respond to standard treatment.[2]

In this case, repeated low dose methylene blue helped in tiding over the fluctuating symptoms due to the release of nitrobenzene from the body stores, without exceeding the maximum dose. Fresh blood transfusion improved the oxygen carrying capacity and haemoglobin content, improving the patient symptomatically. Oral charcoal and purgation up to five days helped to eliminate the body stores of nitrobenzene and prevented secondary deterioration in the patient, as reported in some cases.[1,2] Taking care of nutrition, adequate urine output, and hepatoprotection prevented kidney and liver failure, which have been cited as late effects.[1,6] Forced diuresis led to a rapid fall in methemoglobin levels and improved discolouration.[5] Ascorbic acid supplements are useful for follow-up management of methaemoglobinaemia.[9]

## CONCLUSION

The authors wish to point out that non-availability of intravenous methelene blue[2] should not be a hindrance, as methylene blue powder is available in all gynaecological outpatient departments, and can be made into 1% solution and sterilised in CSSD.
